# Chronic migraine plus medication overuse headache: two entities or not?

**DOI:** 10.1007/s10194-011-0388-3

**Published:** 2011-09-22

**Authors:** Andrea Negro, Paolo Martelletti

**Affiliations:** 1 Department of Medical and Molecular Sciences, Sapienza University, Rome, Italy; 2 Regional Referral Headache Center, Sant’Andrea Hospital, Via di Grottarossa 1035, 00189 Rome, Italy

**Keywords:** Chronic migraine, Refractory chronic migraine, Medication overuse headache, Detoxification, Rehabilitation, OnabotulinumtoxinA

## Abstract

Chronic migraine (CM) represents migraine natural evolution from its episodic form. It is realized through a chronicization phase that may require months or years and varies from patient to patient. The transition to more frequent attacks pattern is influenced by lifestyle, life events, comorbid conditions and personal genetic terrain, and it often leads to acute drugs overuse. Medication overuse headache (MOH) may complicate every type of headache and all the drugs employed for headache treatment can cause MOH. The first step in the management of CM complicated by medication overuse must be the withdrawal of the overused drugs and a detoxification treatment. The goal is not only to detoxify the patient and stop the chronic headache but also to improve responsiveness to acute or prophylactic drugs. Different methods have been suggested: gradual or abrupt withdrawal; home treatment, hospitalization, or a day-hospital setting; re-prophylaxes performed immediately or at the end of the wash-out period. Up to now, only topiramate and local injection of onabotulinumtoxinA have shown efficacy as therapeutic agents for re-prophylaxis after detoxification in patients with CM with and without medication overuse. Although the two treatments showed similar efficacy, onabotulinumtoxinA is associated with a better adverse events profile. Recently, the Phase III Research Evaluating Migraine Prophylaxis Therapy (PREEMPT) clinical program proved that patients with CM, even those with MOH, are the ones most likely to benefit from onabotulinumtoxinA treatment. Furthermore, it provided an injection paradigm that can be used as a guide for a correct administration of onabotulinumtoxinA.

## Introduction

Chronic migraine (CM) constitutes migraine natural evolution from its episodic form. It is realized through a chronicization phase that may require several months or years and varies from patient to patient. The transition to more frequent attacks pattern is influenced by lifestyle, life events, comorbid conditions and personal genetic terrain, and it often leads to acute drugs overuse, rather being accompanied by that.

The International Classification of Headache Disorders, II version revised (ICHD-IIR), includes criteria for CM in which the disorder is defined by headaches on ≥15 days/month for ≥3 months, of which ≥8 days fulfill the criteria for migraine without aura which were successfully treated with acute care medications such as ergots or triptans [1].

Chronic daily headache (CDH) syndromes are a group of headache disorders that occur on ≥15 days/month, for ≥4 h/day for ≥3 months. CM, chronic tension-type headache (CTTH), hemicrania continua (HC) and new daily persistent headache (NDPH) are primary headache disorders, whereas medication overuse headache (MOH) is classified in the ICHD-IIR as an “independent” secondary headache. ICHD-IIR precludes the diagnosis of any of these headache types, other than MOH, if the patient is overusing acute medication [1, 2].

MOH is a common and debilitating disorder, which is characterized by generation, perpetuation and persistence of intense chronic migraine caused by the frequent and excessive use of symptomatic drugs for at least 3 months, for a certain number of days per month [2]. MOH may complicate each type of headache and all the drugs employed for headache treatment can cause MOH.

Classification criteria of abuse are related to the pharmaceutical class applied during acute treatment, namely of 15 days/month for analgesics and non-steroid anti-inflammatory drugs (NSAID), and 10 days/month for mixed drugs (triptans, ergotamines, opioids, and NSAID) [3].

Because of their availability and low cost, barbiturate-containing combination analgesics and over-the-counter caffeine-containing combination analgesics are the greatest problem. Even though triptans overuse headache is not encountered with great frequency, all triptans should be considered potential inducers of MOH [3].

MOH can be distinguished as simple (MOH Type I) or complex (MOH Type II). Simple cases involve relatively short-term drug overuse, relatively modest amounts of overused medications, minimal psychiatric contribution, and no history of relapse after drug withdrawal. In contrast, complex cases often present with multiple psychiatric comorbidities and a history of relapse [4].

## Chronic migraine challenges

The prevalence rate of CM in general population is 2–4% [5]. Each year, approximately 2.5% of patients with episodic migraine (EM) develop new-onset CM [6].

At this time, CM represents the most important challenge for tertiary headache centers, where more than 50% of patients are referred for monitoring the chronicization process and its possible complication with MOH.

Compared with patients with EM, those with CM are female, menopausal, married, unemployed, on polypharmacy, not using oral contraceptives, having worse socioeconomic status, reduced health-related quality of life, increased headache-related burden (including impairment in occupational, social, and family functioning), migraine remission during pregnancy, and having greater psychiatric (e.g., depression, anxiety and chronic pain) and medical comorbidities (e.g., hypertension, diabetes, high cholesterol and obesity) [6, 7].

Among patients in headache clinics or centers of tertiary care, patients with MOH form the largest group along with migraine and tension-type headache. Up to 30% of patients in such centers in Europe, and more than 50% in the USA, present with MOH [8, 9].

MOH affects 1–4% of general population, with prevalence rates similar across different countries [10, 11] but with a higher preponderance in women than in men [11].

It has been noted that the overuse of analgesics for chronic headache is not only prevalent in Europe and North America but also presents in Asian countries [12]. Moreover, clinical evidence demonstrates that overuse associated with chronic forms of headache can occur in childhood and early adolescence and not only in adults and elderly patients [13].

Migraine attacks can increase in frequency over time. Headache experts conceptualize this process with a model that envisions transition into and out of four distinct states: no migraine, low-frequency EM (<10 headaches/month), high-frequency EM (10–14 headaches/month), and CM (≥15 headaches/month) [6]. The transition may be both in the direction of increasing or decreasing headache frequency.

Migraine’s chronicization and the subsequent appearance of MOH are realized through a period of time which involves several months or years. This escalation produces daily, or almost daily pattern, in some cases with symptomatology less adherent to a classic migraine attack [14].

This leads the headache to manifest in a different way from the original headache form since pain can vary according to severity and location. Moreover, the assumption of previously effective medication could induce or worsen headache.

## Roads to migraine chronicization

Patients with an intermediate headache frequency of 6–9 days/month are at greater risk for further progression to CM. The risk is even greater in patients who have headaches on 10–14 days/month [15]. For that reason, in presence of CM’s low prevalence, special attention should be paid to both control and reduction of risk factors which might favor the migraine chronicization process and/or the outbreak of MOH [16].

Risk factors for chronicization can be divided into three categories: nonmodifiable, modifiable, and putative.

Nonmodifiable risk factors include older age, female sex, caucasian race, worse socioeconomic status, low education level [17, 18], and genetic factors [19].

Modifiable factors include attack frequency [17], obesity [20], medication overuse [21], caffeine use/misuse [10, 22, 23], sleep disorders (e.g., snoring, obstructive sleep apnea, insomnia, hypersomnia) [24, 25], stressful life events [26, 27], specific psychological patterns (e.g., depression, anxiety, and personality disorders) [28, 29], behavioral issues [30, 31], and family history of mood disorders and substance use disorders (alcohol, drugs) [32].

Other risk factors currently being investigated include low serum vitamin D levels [33], gastroesophageal reflux disease [34], and proinflammatory and prothrombotic states [14, 35].

Moreover, additional risk for the development of this form of headache comes from wrong conducts, as absence of referral to headache centers during the worsening period, lack of education in avoiding trigger factors, and inadequate life-style rhythms (fasting, sleepiness). Also the recommendations that drugs be taken as early as possible, effective with specific medications like triptans, increases the risk that patients will take more of the drug than is necessary, thus increasing the risk of inducing medication overuse.

Regarding medication overuse, the risk of progression from EM to CM is increased by any use of barbiturates and opioids, while triptans are not associated with the same risk. NSAIDs are either protective or inducers depending on the headache frequency (protective against transition at low-to-moderate monthly headache days, associated with increased risk of transition at high levels of monthly headache days) [36].

Regarding opioids’ association with migraine progression, the effect is dose-dependent (critical dose of exposure: 8 days/month), and more pronounced in men [9, 36]. Barbiturates are also found to induce migraine progression with a dose-dependent effect (critical dose of exposure: 5 days/month) but more pronounced in women [9, 36]. Triptans, on the other hand, induce migraine progression only in those with high migraine frequency at baseline (<14 days/month), but not overall [9, 36]. NSAIDs protect against migraine progression unless individuals have ≥10 headache days/month (when they become inducers, rather than protective), while caffeine containing over-the-counter products increase risk of progression [9, 36].

The substances associated with the overuse have dramatically changed over the past 20 years. There was a significant decrease in the relative frequency of probable ergotamine overuse headache and probable combination analgesic overuse headache, while the frequency of opioids overuse headache remained the same. Conversely, the relative frequency increased significantly for triptans and for combinations of acute medications [37].

## The complex chronic migraine/medication overuse headache should be detoxified first

MOH constitutes a plus of CM and it is hard to think about its appearance not being related to CM itself, unless patients attempt counterproductive stoicism. Since MOH does not stand alone, it should be at least considered a complication of CM and not just a simple form of secondary headache [38].

In MOH sufferers, the treatment of choice is drug withdrawal, which is used by most specialized centers as the primary therapy. The goal of this treatment is not only to detoxify the patients and stop the chronic headache but also to improve responsiveness to acute or prophylactic drugs [39].

Discontinuation of the acute medication can result in worsening of the headache, nausea, vomiting, arterial hypotension, tachycardia, sleep disturbances, restlessness, anxiety, nervousness and rebound headache. Seizures or hallucinations, although rare, are observed in patients who overuse barbiturates containing anti-migraine drugs. These symptoms generally last between 2 and 10 days (average 3.5 days), but can persist for up to 4 weeks [40]. Withdrawal symptoms are usually relieved by further intake of the overused medication, but this could also lead to perpetuation of the overuse.

The withdrawal headache seems to be shorter in patients who have taken triptans (mean 4.1 days) than in patients who have overused ergotamine (mean 6.7 days) or NSAIDs (mean 9.5 days) [41].

A further step beyond drug interruption results upon smoothing symptoms following interruption, through pharmacological support. Treatments for the acute phase of drug withdrawal vary considerably between studies. They generally include fluid replacement, analgesics when strictly necessary for severe rebound headache, tranquillisers, neuroleptics and steroids.

Among all classes of drugs, corticosteroids certainly are the most frequently employed. Oral prednisone constitutes the most common treatment during detoxification [40, 42, 43]. However, studies on the management of withdrawal headache using prednisolone have produced mixed results [44, 45].

Different methods have been suggested for successful drug withdrawal: home treatment, hospitalization, with or without the use of steroids, and with re-prophylaxes performed immediately or at the end of the wash-out period. Even the imparting of advice alone obtained effective drug withdrawal in patients with simple and complicated medication overuse headache [46].

Currently, there are no universally accepted standardized therapeutic protocols and no specific guidelines for controlled trials in MOH. An agreement has been reached only for withdrawal from abuse as *conditio sine qua non* to reinstate the natural course of CM or high-frequency migraine [47].

### Inpatient versus outpatient drug withdrawal

Between the different drugs withdrawal strategies, inpatient withdrawal seems the most helpful, and should be preferred in patients who take barbiturates, in those who are not able to stop taking medications as outpatients and also in those with high levels of depression [48]. Conversely, an outpatient treatment can be an alternative for highly motivated and self-disciplined patients who take a single drug or analgesic not containing barbiturates, and who do not have a high level of depression or anxiety [44, 48–50].

A direct comparison between inpatient and outpatient withdrawal treatment shows that both methods lead to a significant reduction of headache days/month and migraine disability after 12 months, without superiority of one method [51]. Since the outpatient withdrawal approach is less expensive than the inpatient approach, and is as successful in motivated patients, it is the preferred choice in many cases [40, 43].

Another helpful alternative for drug withdrawal is infusion therapy within a day-hospital setting, limiting patient’s permanence in hospital at a maximum of 6 h during the infusion therapy [51].

A withdrawal and detoxification therapeutic regimen, which utilized abrupt discontinuation of the overused drug and a therapeutic protocol including i.v. hydration, dexamethasone, metoclopramide and benzodiazepines for 7–10 days, obtained satisfactory results at 6 months of follow-up in a sample of patients suffering from probable CM and probable MOH during admission in eight Italian hospitals. In this case, prophylactic medication was started immediately after admission [52].

Data suggest that the patients affected by CDH and medication overuse benefit from withdrawal therapy performed during hospitalization (dexamethasone 4 mg i.v./day for 1 week, diazepam 6 mg/day for 10 days) along with prophylaxis [53].

### Abrupt versus gradual withdrawal

Although there are no studies comparing gradual and abrupt interruption, the widespread opinion of specialists considers drug withdrawal to be more effective when done abruptly because this is believed to achieve a fast resolution of the drug-induced pain-coping behavior [21, 40, 43, 49, 54].

Most drugs causing MOH can be stopped abruptly. This is most particular to the overuse of triptans, ergots, paracetamol, aspirin and NSAIDs. However, due to the possibility of severe withdrawal symptoms, gradual withdrawal is appropriate with opioids, barbiturates and, in particular, benzodiazepines [54]. As with drugs that produce a withdrawal syndrome, gradual reduction in caffeine intake may be preferable to abrupt withdrawal [55].

### Risk of relapses

The treatment can be considered successful when clinical improvement is confirmed after at least 1 year of follow-up after withdrawal.

Findings from the recent studies suggest that patients have a greatest risk for relapse within the first 12 months but have a decreased risk of relapse when they have avoided medication overuse for 12 months after withdrawal therapy [56].

Relapse percentages during the first year after withdrawal range between 22 and 44% [52]. Other three studies considering a longer observation period (9–35 months) [50, 57, 58] recorded success rates of 60, 70 and 73%, respectively. Studies with a longer follow-up period (4–6 years) found relapse rates between 40 and 60% [56, 59–61].

Reported risk factors for relapse include: male gender [52]; TTH or a combination of migraine plus TTH, rather than migraine alone [59]; frequency of primary headache disorder [57]; longer duration of migraine with more than eight headache days/month [59]; long duration of migraine before medication overuse [62]; long duration of drug overuse [62]; greater number of the previous preventive treatments [59, 62]; intake of combined analgesic drugs (e.g., combination of one or more NSAIDs with caffeine or codeine) [52, 59]; use of codeine-containing drugs [63]; ergotamine or triptan withdrawal more than analgesic withdrawal [57, 59]; using the causative medication again after withdrawal therapy [44]; lower improvement after drug withdrawal [62]; low sleep quality [63]; high body pain as measured by the Short Form 36 (SF-36) questionnaire [63]; and disability score for chronic headache estimated by Migraine Disability Assessment Score (MIDAS) [57].

## Chronic migraine re-prophylaxis after detoxification

Although according to estimates about 25% of all migraine sufferers should be offered preventive therapies, prophylactic medications are still significantly underutilized [64].

A preventive treatment should be considered for all patients with ≥3 disabling attacks/month that fail to adequately respond to acute medication, and migraines that greatly interfere with activities of daily life despite appropriate use of acute medications [65].

The primary goals of preventive migraine therapy are to reduce the frequency and severity of attacks, to reduce reliance upon acute medications, to reduce visits to the emergency room or doctor’s surgery and to improve the migraine patient’s quality of life [65].

A long-term preventive treatment should be encouraged in those patients at risk for migraine chronicization, with medication overuse or contraindication to acute therapies. A study conducted in a large series to assess prospectively the impact of prophylaxis on health-related quality of life (HRQOL), using the SF-36 questionnaire, and daily activities, using MIDAS, indicates that migraine prophylaxis has the potential to reduce the global burden of migraine on individuals and society [66].

## Topiramate and onabotulinumtoxinA: two options, but one choice

Up to now, only topiramate and local injection of onabotulinumtoxinA have shown efficacy as therapeutic agents for re-prophylaxis after detoxification in patients with CM with and without medication overuse.

The use of topiramate in preventing migraine’s chronicization process or in reverting consolidated CM is well known.

Topiramate proved to be effective in reducing migraine headache days [67, 68] and able to reduce the risk of transformation to a chronic form [69]. The most common adverse events (AEs) during topiramate treatment are paresthesias (8.0%), cognitive symptoms (7.3%), fatigue (4.7%), insomnia (3.4%), nausea (2.3%), loss of appetite, anxiety, and dizziness (2.1%) [70]. These side effects are not known in association with onabotulinumtoxinA [71].

Two studies compared the efficacy and safety of topiramate and onabotulinumtoxinA prophylactic treatment in patients with CM [72, 73]. Significant within, but not between-groups, improvements were observed for several outcomes: treatment responder rate; mean change from baseline in number of headache/migraine days/month; headache/migraine-free days/month; days on headache medication; average severity of headache/migraine episodes/month; clinical improvements in quality of life, sleep, work and recreational activities; Headache Impact Test (HIT); and MIDAS.

Although topiramate and onabotulinumtoxinA resulted in similar efficacy in these studies, the two treatments resulted in different AE profiles. The overall discontinuation rate was significantly higher in the topiramate than in the onabotulinumtoxinA group, with AEs being the primary reason for withdrawal in the topiramate group [72, 73]. The results of these studies are in accordance with controlled trials in the CM population that have reported discontinuation rates of 25–44.2% with topiramate compared with 10–25% with onabotulinumtoxinA [67, 68, 74–76].

The safety profile indicates that onabotulinumtoxinA is safe and well tolerated in the CM population, with a few patients discontinuing treatment due to AEs (1.4–3.8%) [75–79].

Given the substantial AEs and adherence issues associated with available pharmacotherapies for CM, the relatively mild AEs associated with onabotulinumtoxinA may present an attractive treatment alternative.

Results of two randomized, double-blind, placebo-controlled trials have provided further insight into which patients, dosages, and injection protocol may yield the best results from prophylactic onabotulinumtoxinA therapy [75, 76].

These trials have been the guidance for the study design and the injection paradigm of the Phase III Research Evaluating Migraine Prophylaxis Therapy (PREEMPT) clinical program. This consists in two phase 3, multicenter double-blind, parallel-group, placebo-controlled studies (PREEMPT 1 and 2) that evaluated the efficacy of onabotulinumtoxinA (155–195 U) in 1,384 adult patients with CM who have 50% or more headache days fulfilling migraine or probable migraine criteria and have four or more distinct headache episodes at baseline screening [77–79].

Important end points (primary and secondary) were reduction of frequency of headache days (primary in PREEMPT 2 and pooled analysis; secondary in PREEMPT 1) and headache episodes (primary in PREEMPT 1; secondary in PREEMPT 2 and pooled analysis).

OnabotulinumtoxinA resulted significantly more effective than placebo in terms of frequency reduction during headache days in both PREEMPT 1 (*P* = 0.006) and PREEMPT 2 (*P* < 0.001) [78, 79]. Statistically significant improvement from baseline after onabotulinumtoxinA compared with placebo treatment was seen for headache episodes in PREEMPT 2 (*P* = 0.003) [79], but not in PREEMPT 1 [78]. Pooled analysis demonstrated that onabotulinumtoxinA treatment significantly reduced mean frequency of headache days (−8.4 onabotulinumtoxinA, −6.6 placebo; *P* < 0.001) and headache episodes (−5.2 onabotulinumtoxinA, −4.9 placebo; *P* = 0.009) [77].

Statistically significant improvements with onabotulinumtoxinA were seen in a number of secondary outcome measures, including: migraine days (*P* < 0.001); reductions in moderate or severe headache days (*P* < 0.001); cumulative hours of headache on headache days (*P* < 0.001); headache episodes (*P* < 0.009); migraine episodes (*P* < 0.004); acute medication use (*P* < 0.001); and proportion of patients with a severe disability (*P* < 0.001) [77]. In PREEMT, onabotulinumtoxinA was also reported to be effective in a subgroup of patients with medication overuse. Furthermore PREEMPT showed a good safety profile. AEs occurred in 62.4% of the onabotulinumtoxinA group and 51.7% of the placebo group. Most AEs were mild-to-moderate in severity, and a few patients discontinued the trial due to AEs (onabotulinumtoxinA, 3.8%; placebo, 1.2%). The only AE reported with an incidence over 5% in the onabotulinumtoxinA group were neck pain (8.7%) and muscular weakness (5.5%) [77–79].

PREEMPT trials have two major achievements: first of all, they prove that patients with CM, even those who overuse acute headache medication, are an appropriate target group supporting the previous studies which identified CM patients as the ones most likely to benefit from onabotulinumtoxinA treatment [75, 76]; second, these studies provide an injection paradigm [combination of Fixed Sites Fixed Doses (155 U) plus “eventually” Follow The Pain (40 U) protocols] that can be used as a guide for a correct administration of onabotulinumtoxinA [80].

### Concluding debates on onabotulinumtoxinA and CM

Despite these data, PREEMPT trials received several critics, particularly regarding the diagnosis of CM (65% of patients had medication overuse, which precludes the diagnosis of CM according to the IHS), the high percentage (35%) of patients that never before received any pharmacological prophylaxis, and the effectiveness of blinding (*sine* onabotulinumtoxinA weakens muscles and changes the facial expression while placebo does not) [81–83]. Even the injection protocol adopted received critics [84].

However, the phase of the protracted debate on the efficacy of onabotulinumtoxinA in CM patients must be considered ended. In 2010, on the basis of the results of PREEMPT clinical program, first the Medicines and Healthcare Products Regulatory Agency (MHRA) in the UK, and later the Federal Drug Administration (FDA) in USA, approved onabotulinumtoxinA injection therapy for the prevention of headaches in adults with chronic migraine.

## Identify the right targeting: not all patients are the same

Patient selection appears to be a key to the successful use of the toxin in headache management.

The pharmacologic profile of onabotulinumtoxinA makes it an appealing candidate for CM patient’s profile. Its long duration of action (3 months on average) and favorable adverse effect profile makes it particularly attractive for patients with poor compliance, adherence, or AE profile with oral preventive medications [85].

Overall, several studies show a more favorable response to onabotulinumtoxinA in: CM patients with unilateral headache, scalp allodynia, and pericranial muscle tenderness [86, 87]; CDH patients with a shorter disease duration (<30 years) [88]; CDH patients who did not overuse pain medications than those who did [86]; patients who do not respond effectively to any of the preventative treatments (chronic refractory migraine) [86].

Furthermore onabotulinumtoxinA prophylactic therapy markedly decreases costs related to acute headache medication use suggesting onabotulinumtoxinA as a cost-reasonable option for medication offsets alone especially in patients with chronic headache with higher acute medication use [89, 90].

## Keys to ideal management

Despite MOH is a disorder characterized by very own features outlining a peculiar and autonomous disease, it should be more correctly considered a complication of CM, if not even in some cases its natural evolution.

The first steps in the management of CM complicated by medication overuse must be the withdrawal of the overused drugs and a detoxification treatment; that in order to stop the chronic headache and mostly to improve the answer to the second step of the management. It is represented by re-prophylaxis with preventive medications that must be started immediately after the detoxification.

For its safety profile and proven efficacy onabotulinumtoxinA is the best therapeutic option and the first preventive medication to choose in CM patients, also in those already underwent to detoxification for medication overuse.

Decades of research on the individual clinical features of CDH sufferers, and on their response to the medications currently available, have demonstrated the existence of a subgroup of patients with a CDH which appeared to be “resistant” to treatment.

Even if “resistant CDH” is far from adding to the international classification of headache disorders, physicians should think of this when they face a migraine patient.

This is especially true for whom working in headache clinics or centers of tertiary care, which are turning more often in patients with severe forms of CM.

Thus, the future in the treatment and relapse prevention of CM complicated by MOH, and mostly of “resistant CDH”, consists in considering how drugs currently used, such as triptans and emerging therapies, present responsivity profiles related to well-defined genetic polymorphisms [19, 91, 92]. The feasible diagnostic setting for a tailored treatment of CM based on the application of pharmacogenomics will allow us to predetermine the efficacy of single old and new drugs by avoiding abuse and chronicization due to non-responsivity of the abused drug.
